# A Computational Study of the Hydrodynamics in the Nasal Region of a Hammerhead Shark (*Sphyrna tudes*): Implications for Olfaction

**DOI:** 10.1371/journal.pone.0059783

**Published:** 2013-03-29

**Authors:** Alex D. Rygg, Jonathan P. L. Cox, Richard Abel, Andrew G. Webb, Nadine B. Smith, Brent A. Craven

**Affiliations:** 1 Department of Mechanical and Nuclear Engineering, The Pennsylvania State University, University Park, Pennsylvania, United States of America; 2 Applied Research Laboratory, The Pennsylvania State University, University Park, Pennsylvania, United States of America; 3 Department of Chemistry, University of Bath, Bath, United Kingdom; 4 Department of Mineralogy, Natural History Museum, London, United Kingdom; 5 C. J. Gorter Center for High Field MRI, Department of Radiology, Leiden University Medical Center, Leiden, The Netherlands; 6 Department of Bioengineering, The Pennsylvania State University, University Park, Pennsylvania, United States of America; Hospital Nacional de Parapléjicos - SESCAM, Spain

## Abstract

The hammerhead shark possesses a unique head morphology that is thought to facilitate enhanced olfactory performance. The olfactory chambers, located at the distal ends of the cephalofoil, contain numerous lamellae that increase the surface area for olfaction. Functionally, for the shark to detect chemical stimuli, water-borne odors must reach the olfactory sensory epithelium that lines these lamellae. Thus, odorant transport from the aquatic environment to the sensory epithelium is the first critical step in olfaction. Here we investigate the hydrodynamics of olfaction in *Sphyrna tudes* based on an anatomically-accurate reconstruction of the head and olfactory chamber from high-resolution micro-CT and MRI scans of a cadaver specimen. Computational fluid dynamics simulations of water flow in the reconstructed model reveal the external and internal hydrodynamics of olfaction during swimming. Computed external flow patterns elucidate the occurrence of flow phenomena that result in high and low pressures at the incurrent and excurrent nostrils, respectively, which induces flow through the olfactory chamber. The major (prenarial) nasal groove along the cephalofoil is shown to facilitate sampling of a large spatial extent (i.e., an extended hydrodynamic “reach”) by directing oncoming flow towards the incurrent nostril. Further, both the major and minor nasal grooves redirect some flow away from the incurrent nostril, thereby limiting the amount of fluid that enters the olfactory chamber. Internal hydrodynamic flow patterns are also revealed, where we show that flow rates within the sensory channels between olfactory lamellae are passively regulated by the apical gap, which functions as a partial bypass for flow in the olfactory chamber. Consequently, the hammerhead shark appears to utilize external (major and minor nasal grooves) and internal (apical gap) flow regulation mechanisms to limit water flow between the olfactory lamellae, thus protecting these delicate structures from otherwise high flow rates incurred by sampling a larger area.

## Introduction

The olfactory chamber of the hammerhead shark is an extremely complex organ that to date has largely been studied on a morphological basis. Such studies [1]–[3] have provided insight into how different anatomical structures may contribute to olfaction. Externally, one of the most distinguishable features of the Sphyrnidae family is the broad, flat head known as a cephalofoil. This unique head morphology provides a wide lateral separation between olfactory organs that may be used by the shark to resolve spatial gradients in odorant concentration, resulting in enhanced bilateral sampling for olfactory tropotaxis [2], [4], [5]. Additionally, the cephalofoil of the hammerhead shark contains a narrow groove, termed the prenarial groove (or major nasal groove [3]), that extends medially from the incurrent nostril and is thought to direct flow towards the inlet naris, thereby permitting the shark to sample a larger volume of fluid [2], [3]. Abel et al. [3] also described the presence of a minor nasal groove, located anterior and parallel to the incurrent nostril, that may also direct flow toward the inlet naris while regulating the amount of flow entering the olfactory chamber, thereby protecting the fragile olfactory lamellae.

Internally, the olfactory organ of the hammerhead shark contains numerous lamellae that increase the surface area of the olfactory sensory epithelium [1]–[3]. The lamellae are stacked in parallel and arranged in two separate rows, consisting of pairs of dorsal and ventral lamella that are attached to either the dorsal or ventral wall of the nasal chamber, respectively, and separated by a central support known as a raphe [3], [6]. Kajiura et al. [2] found that sphyrnid shark species generally possess a greater number of olfactory lamellae than carcharhinid species. For example, *Sphyrna tudes* possesses 90–100 pairs of lamellae compared with approximately 60 and 40 for *Carcharhinus plumbeus* and *Scoliodon laticaudus*, respectively [2], [3]. Even so, sphyrnid shark species do not possess significantly more lamellar surface area compared to carcharhinid species [2]. Thus, although the cephalofoil of the hammerhead shark may provide external hydrodynamic advantages, it apparently does not provide increased surface area for olfaction compared to other species of sharks. However, factors other than sensory surface area may significantly influence olfactory acuity, e.g., internal hydrodynamics and odorant mass transport phenomena. Thus, a proper description of the hydrodynamics of olfaction is required in understanding the mechanisms that contribute to olfactory acuity.

Abel et al. [3] conducted the first hydrodynamic flow visualization experiment in the nasal region of a hammerhead shark (*Sphyrna tudes)* using a reconstructed life-sized plastic model of the head placed in a water tunnel. This study revealed some features of the external hydrodynamics and showed significant gross circulation of fluid through the olfactory chamber. In addition, the experiments investigated the effects of changing the oncoming flow angle, as hammerheads are known to sweep their heads in an arc as they swim [7]. However, this study, which used a reconstructed model of low resolution, was unable to quantify the detailed internal hydrodynamics.

The objective of this study was to reconstruct an anatomically-accurate, three-dimensional model of the head and olfactory chamber of *Sphyrna tudes* from high-resolution X-ray micro-computed tomography (micro-CT) and magnetic resonance imaging (MRI) scans of a cadaver specimen. This includes the numerous lamellae that fill the olfactory chamber. Using this reconstructed model, high-fidelity computational fluid dynamics (CFD) simulations were carried out to study the external and internal hydrodynamics of olfaction in the hammerhead shark during swimming.

## Methods

### Specimen

The *Sphyrna tudes* specimen used in this study was loaned with permission from the Natural History Museum, London. It has been in preservation since 1959 in a solution of 70% methylated spirits and 30% distilled water. The specimen consists of the head and part of the gill region. The sex of the specimen is unknown, but its total length is estimated to be approximately 90 cm [3]. Additional details regarding the specimen can be found in the related study by Abel et al. [3].

### Micro-CT Acquisition

After removal of the specimen from the preservative, it was mounted for X-ray scanning in a cling film-covered recess cut from a block of florist’s foam. Each olfactory chamber was emptied of preservative prior to the scan. Scanning was performed using a HMXST 225 CT system (Nikon Metrology, Tring, UK). The X-rays were generated from a tungsten target using a voltage and current of 180 kV and 105 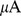, respectively. A total of 3,142 angular projections were collected at 0.1146° intervals in a single 360° rotation. The radial projections were reconstructed into a three-dimensional matrix of 1,897×1,830×630 (L×W×H) 124.5 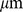 cubic voxels using the software package CT-Pro (Version 2.0, Nikon Metrology, Tring, UK).

### MRI Acquisition

For MRI scanning, the cadaver specimen was placed in a flexible plastic container that was filled with degassed water. Trapped air was minimized by applying a vacuum to the container. The MRI scan was acquired on a Philips Achieva whole body 7 Tesla system, with a 58 cm diameter clear bore. The gradients have a maximum value of 40 mT/m with a slew rate of 200 mT/m/s. The transmit head coil (NM-008A-7P, Nova Medical, Wilmington, MA) is an actively-detunable quadrature birdcage, with sixteen elements (each 2.5 cm wide), an inside diameter of 29.2 cm, outside diameter of 37.5 cm, and a physical length of 26 cm. The 16-channel receive phased array (NMSC025-16-7P, Nova Medical, Wilmington, MA) consists of eight radially-gapped rows of z-overlapped coil pairs, with an inner diameter of 25.5 cm. The advantage of the whole-body scanner, compared to using a smaller animal scanner (e.g., [8]), was that the entire specimen could be scanned in one experiment. A multiple slice spin-echo experiment was performed with the following data parameters: time-of-repetition (TR) 3 s, time-to-echo (TE) 12 ms, in-plane data matrix 880×880, in-plane spatial resolution 150×150 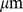, slice thickness 350 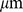, 69 slices, 20 signal averages, and a total data acquisition time of approximately 14 hours.

### Surface Reconstruction

**Figure 1:**
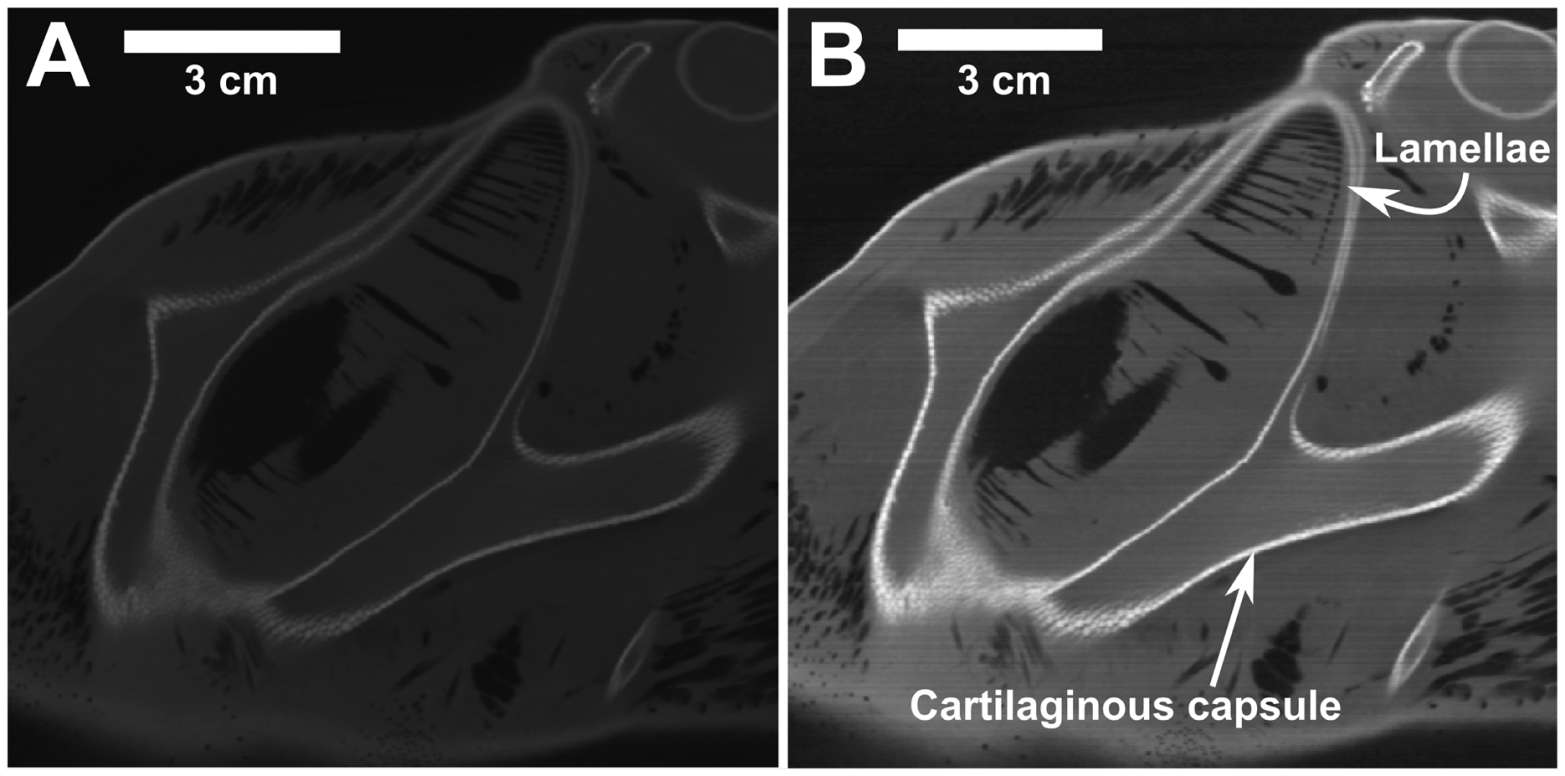
Effect of image processing on the raw CT data. Comparison of a (A) raw and (B) processed CT slice.

Image processing of the raw CT and MRI data yielded a high-contrast data set having a sharp distinction between the tissue and nasal passages, which is optimal for image segmentation. Using custom image processing software written in MATLAB (MathWorks, Inc., Natick, MA, USA) that was also used by Craven et al. [8] and Holmes et al. [9], several operations were performed to remove noise and improve image contrast. First, a 3×3 median filter was applied to each data set, thereby removing noise while preserving edges. A linear contrast stretch was then used to improve the contrast between the light and dark areas within the image, making it easier to distinguish the tissue and nasal passages. Finally, to ensure uniform contrast between consecutive image slices, a controlled saturation was used, where a small percentage of the brightest pixels in each slice were saturated based on a histogram analysis of the gray level intensities. As shown in Figure 1, this resulted in a uniform, high-contrast data set, enhanced for image segmentation.

**Figure 2:**
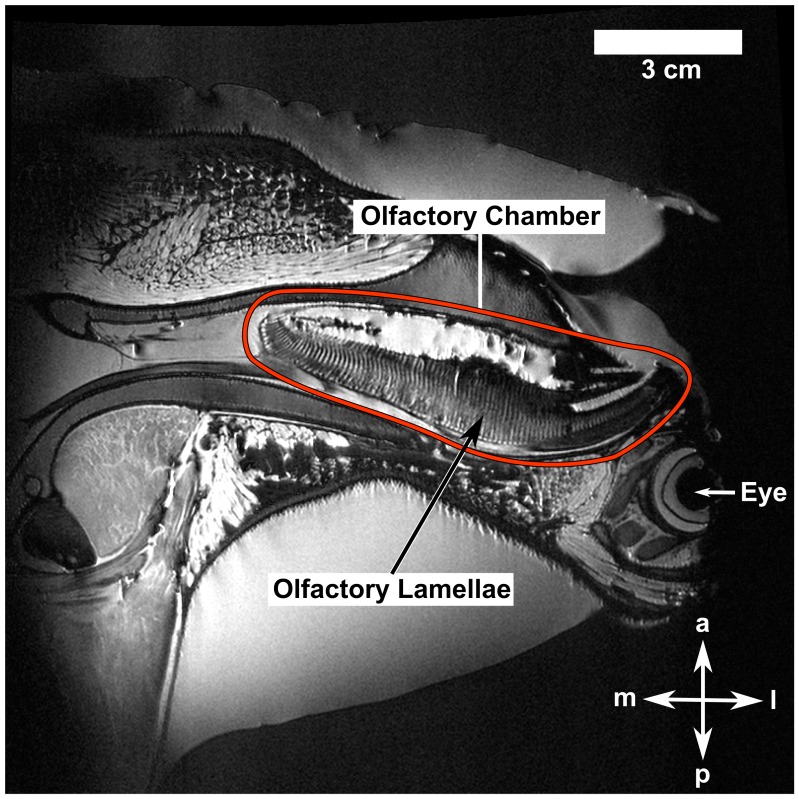
Sample MRI data. Slice of the MRI data, illustrating the separated olfactory lamellae in the right olfactory chamber of the hammerhead shark (*Sphyrna tudes*). a: anterior; l: lateral; m: medial; p: posterior.

The image segmentation process consisted of partitioning the CT data into its constituent regions, i.e. tissue, nasal chamber, and sensory epithelium. Assuming bilateral symmetry, this process was performed on the right olfactory chamber alone. Segmentation of the large nasal passages was accomplished using automated thresholding. As a result, very little manual intervention was required in these regions. However, despite the preparatory image processing, there were many regions in the processed CT data where the tissue-nasal passage interface could not be reliably distinguished. This was primarily due to the fact that many of the olfactory lamellae were “clumped” together. As noted in Abel et al. [3], this clumping was due to the fact that the CT scans were acquired in air. As such, manual segmentation of the lamellae was performed using the MRI scan of the specimen as a reference, which was acquired in degassed water (avoided clumping of the lamellae) and was of adequate resolution to resolve the individual lamellae, as shown in Figure 2.

**Figure 3:**
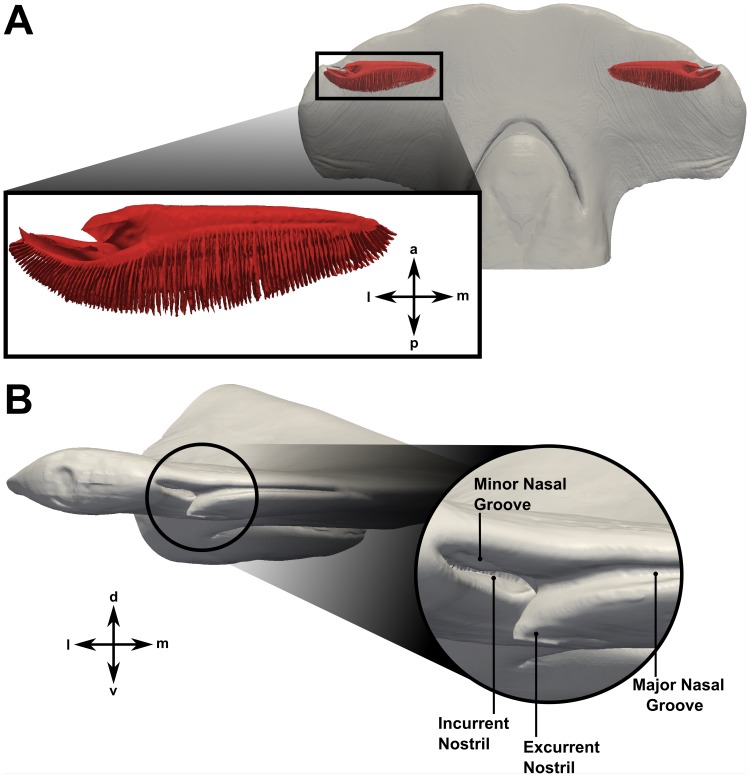
Reconstructed surface model of the head and olfactory chamber of *Sphyrna tudes*. (A) Surface model reconstructed from high-resolution CT and MRI scans of the hammerhead shark. The olfactory chamber is shaded red. (B) Close-up of the surface model, highlighting the external morphology of the nasal region. a: anterior; d: dorsal; l: lateral; m: medial; p: posterior; v: ventral.

These segmented data were then used to generate a three-dimensional surface model of the head and olfactory chamber of *Sphyrna tudes*, including the numerous olfactory lamellae, via a modified form of the marching cubes algorithm [8], [10]. Minor smoothing of the reconstructed surface model was performed to reduce surface “staircasing. ” Using a Laplacian smoothing algorithm, this process was carried out such that the internal volume of the original structure was preserved. A comparison of the original and smoothed models showed a 0.9% difference in internal volume. Figure 3A shows the final reconstructed model, with the olfactory chamber highlighted in red. Figure 3B illustrates the significant external morphological features in the nasal region, including the incurrent and excurrent nostrils and the major and minor nasal grooves.

**Figure 4:**
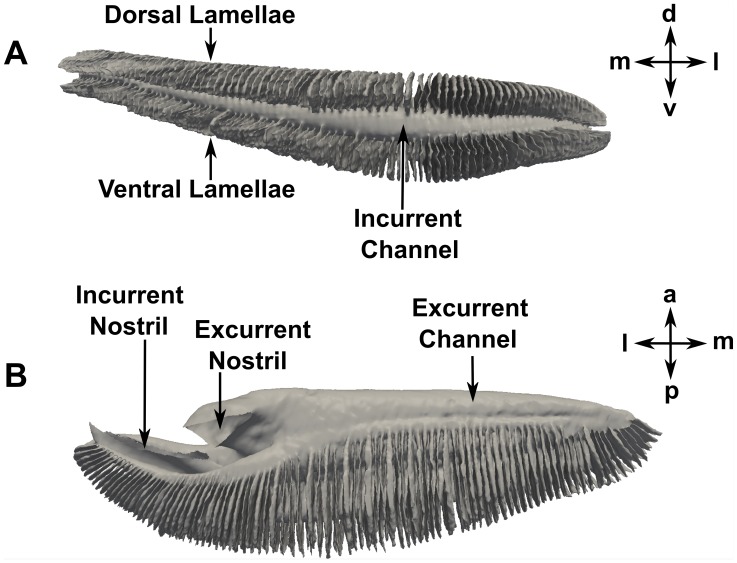
Three-dimensional anatomy of the right olfactory chamber. (A) Posterior view; (B) Ventral view. a: anterior; d: dorsal; l: lateral; m: medial; p: posterior; v: ventral.

The three-dimensional anatomy of the olfactory chamber is illustrated in Figure 4. Functionally, the incurrent and excurrent nostrils serve as the entrance and exit to the olfactory chamber, respectively. Internally, the incurrent and excurrent channels, which are parallel to one another, form a U-shaped channel that feeds the channels between adjacent lamellae (the sensory channels). Approximately 90 pairs of lamellae (nominal gap width of ∼0.25 mm) fill the olfactory chamber, providing 79.7 cm^2^ of total lamellar surface area. Based on descriptions of the distribution of sensory and nonsensory epithelium in the shark olfactory organ [3], [11]–[13], the sensory surface area in the present specimen was calculated to be approximately 60 cm^2^ (75% of the total surface area). Unfortunately, neither the micro-CT or MRI scans resolved any secondary folds on the lamellae and, thus, these folds are not included in the present model or in the calculations of epithelial surface area. However, the influence of these microscale structures is a topic of future work, where we plan to acquire high-resolution scans of the olfactory lamellae in order to characterize the microstructural morphology and its influence on the macroscale hydrodynamics and transport phenomena.

**Figure 5:**
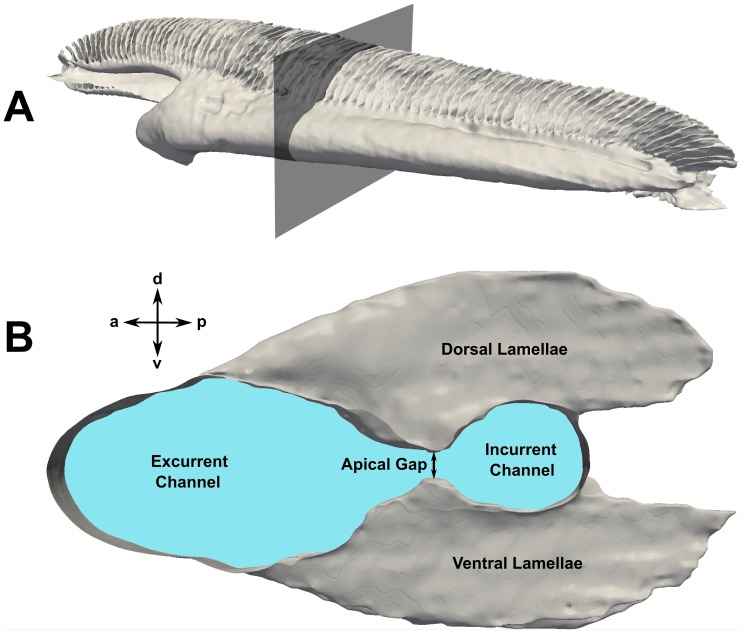
Location of the apical gap. At the location indicated in (A), the apical gap (located between the dorsal and ventral lamellae) is illustrated in a section of the olfactory chamber (B). The blue shading indicates the wetted area of the olfactory chamber that is in contact with water. a: anterior; d: dorsal; p: posterior; v: ventral.

In reconstructing the olfactory chamber, the gap between the apical portions of the dorsal and ventral lamellae, termed the “apical gap” [3], was preserved in the anatomical model. As discussed by Abel et al. [3], the gap may conceivably have arisen in the present specimen as a result of clumping of the olfactory lamellae when the micro-CT scan was acquired in air, or due to postmortem shrinkage of the lamellae caused by preservation of the specimen [14]. However, the gap was clearly present in the MRI data, which was acquired in degassed water and avoided clumping of the lamellae. Further, a number of studies indicate that the apical gap may exist *in vivo* and serve a significant hydrodynamic function. For example, several studies [1], [2], [11], [15] include illustrations of hammerhead olfactory lamellae, where an apical gap can be observed. Most convincingly, however, Schluessel et al. [11] provides photographs that depict the olfactory lamellae of 21 different species of elasmobranchs, where the apical gap is clearly present in *Sphyrna lewini* and is absent in six other species, indicating that the apical gap is likely not a result of postmortem shrinkage or some other factor. Furthermore, Døving et al. [16] observed in a tench and eel that, at high flow velocities, an internal “shunt” was created that allowed fluid to bypass the sensory channels. In terms of hydrodynamic function, Theisen et al. [13] and Zeiske et al. [12] both hypothesize that this shunt mechanism (e.g., the apical gap) could be used to regulate flow through the sensory channels and thus protect the delicate lamellae. In *Sphyrna tudes*, the presence of an apical gap may play a similar physiological role. Thus, based on evidence in the literature, and the presence of the gap in both the micro-CT and MRI scans of the present specimen, the apical gap was preserved in the reconstructed three-dimensional model of the olfactory chamber (see Figure 5).

### Mesh Generation

**Figure 6:**
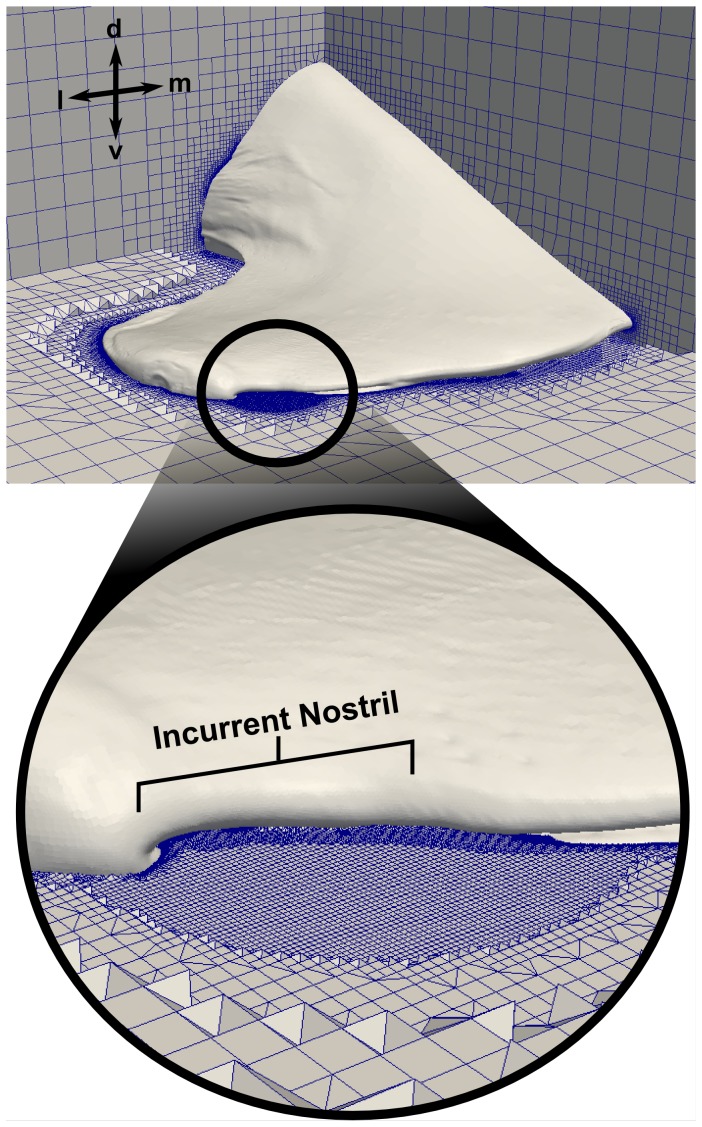
External computational mesh. The external computational mesh for the fine CFD model, shown with an inset illustrating the spherical refinement region encompassing the incurrent and excurrent nostrils. d: dorsal; l: lateral; m: medial; v: ventral.

**Figure 7:**
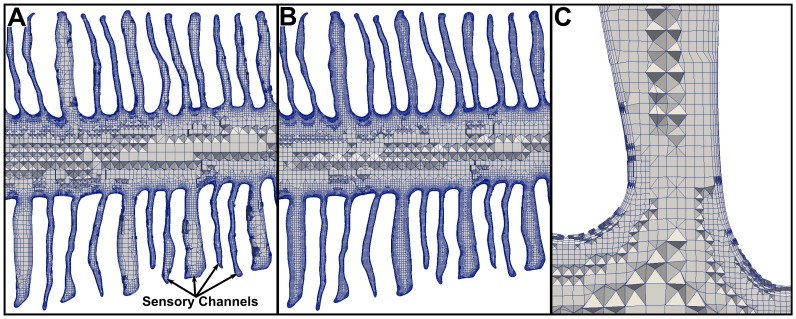
Internal computational mesh. Comparison of the internal mesh resolution for the (A) coarse and (B) fine CFD models within a section of the nasal passages. Panel (C) shows the near-wall mesh in a representative sensory channel for the fine CFD model.

High-fidelity computational meshes were generated from the smoothed, reconstructed surface model using the hexahedral-dominant, unstructured mesh generation utility, snappyHexMesh, available in the open-source computational continuum mechanics library, OpenFOAM. Two meshes of different resolutions (coarse and fine) were generated in order to carry out a CFD mesh refinement study. The coarse mesh contained approximately 36 million computational cells, while the fine mesh consisted of roughly 72 million cells. Figure 6 shows the external resolution for the fine mesh, which included a spherical refinement region encompassing the incurrent and excurrent nostrils to resolve flow entering and exiting the olfactory chamber. Internally, significant refinement was required to resolve flow within the small sensory channels. The coarse mesh contained roughly 13 computational cells across a typical sensory channel, while the fine mesh contained approximately 17 cells across a channel. Figure 7 illustrates the internal resolution of the coarse and fine meshes in the vicinity of the lamellae. Both meshes included several wall-normal layers along the internal surfaces of the olfactory chamber in order to accurately capture large, near-wall velocity gradients.

### Assumptions

The CFD simulations assume that the external and internal anatomy of the reconstructed model is rigid, an assumption that is well-founded with perhaps the exception of the olfactory lamellae. Structurally, each lamella is comprised of two layers of epithelium separated by connective tissue, which is attached to the wall of the nasal chamber and to a central support known as a raphe [3], [6]. Though these attachment points provide structural support along most of the boundary, approximately one-third of the boundary is unsupported and free to deflect since the connective tissue permits some flexibility. Such flexibility is evident from the CT scans of the specimen that were acquired in air (see Figure 1), where clumping of the lamellae occurs due to surface tension and the inability of the lamellae to support their own weight. However, when the olfactory organ is filled with water, the influence of surface tension is reduced and the lamellae are further supported by the dense liquid. Consequently, as shown in the MRI scans that were acquired in water (see Figure 2), the lamellae are freestanding and separated from one another, forming a parallel lamellar array that is likely to be the hypothetical *in vivo* state of the lamellae under stationary, quiescent flow conditions (i.e., when there is no flow through the olfactory organ, a hypothetical state since hammerhead sharks must continuously swim to breathe).

Functionally, when the hammerhead swims and water flows through the olfactory chamber, if a pressure difference is induced between adjacent sensory channels, then a force will be exerted on the intervening lamella that may cause it to deflect. Unfortunately, this cannot be assessed *a priori* since the pressure distribution in the olfactory chamber is unknown. Thus, an *a posteriori* analysis was conducted, which revealed that pressure differences across lamellae are ∼10–50 Pa at the fastest simulated swimming speed (1.55 m/s). Given a lamellar cross-sectional area of ∼0.1 cm^2^, a pressure force of ∼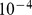 N is exerted on each lamella. Unfortunately, the material properties of the lamellae are unknown, which precludes calculation of the resulting deflection. But, given that only one-third of the boundary is unsupported, we anticipate that such a small, distributed pressure force is unlikely to yield significant deflections *in vivo*. Even so, obtaining high-resolution scans of the olfactory lamellae and characterizing the microstructural morphology and material properties is a topic of future work that will further elucidate the *in vivo* state and flexibility of the lamellae. Given such data, and the present CFD model, future fluid-structure interaction (FSI) simulations may be carried out to quantify the extent of lamellae deflection *in vivo*.

Additionally, the thin viscous mucus layer that covers the sensory epithelium [17]–[20] is assumed to have a negligible effect on the internal hydrodynamics, similar to the assumption that the nasal mucus layer in mammals has a negligible influence on the internal fluid dynamics [21]–[23]. Specifically, the thickness of the olfactory mucus layer in fish is likely to be on the order of the length of the cilia in the olfactory epithelium [19], which are 3–8 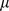
m long in neoselachians [20]. Given an interlamellar gap width of ∼250 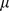
m, the olfactory mucus layer is less than 5% of the sensory channel width. Further, studies of mucus properties reveal that the glycoproteins in mucus give it the consistency of a viscoelastic gel [19], [24]–[26]. Thus, it is reasonable to assume that the viscosity of olfactory mucus will be significantly larger than that of seawater. Taken together, the viscoelastic properties and the small relative thickness of the mucus layer justify the specification of no-slip boundary conditions on all solid surfaces of the head and olfactory chamber.

Furthermore, any motile cilia present in the olfactory epithelium are assumed to have a negligible effect on the macroscale hydrodynamics. Motility of sensory and non-sensory cilia in the olfactory epithelium of fish has been reported [16], [18], [27], [28], with some evidence to suggest ciliary metachronism (coordinated movement) [20]. However, given their small size relative to the sensory channel width and the viscous mucus lining, any type of coordinated or uncoordinated cilia movements are unlikely to affect the macroscale hydrodynamics. Rather, mucus-propelling motile cilia likely play a crucial role in protecting the sensory epithelium by clearing foreign matter [20], as they do in mucociliary clearance of inhaled contaminants in the mammalian respiratory tract. In this case, the flow of mucus is ∼1 cm/min [29], [30], much slower than the anticipated flow speeds within the olfactory organ of the hammerhead shark.

Since the olfactory chamber is passively ventilated as the shark swims [3] (as opposed to pulsatile flow induced by breathing [9]), steady swimming conditions are investigated here. Further, the present CFD simulations assume that the shark’s head is stationary, with the body axis parallel to the oncoming flow stream (i.e., 0° yaw angle). Though hammerhead sharks are known to swing their head from side to side as they swim [7], thereby changing the oncoming flow angle, this occurs at relatively low frequencies (0.6–0.8 Hz) [3]. Thus, we do not expect this movement to significantly affect the overall hydrodynamics in the nasal region. Consequently, steady-state solutions of the governing Navier-Stokes equations are presented in this study. Future transient CFD simulations that include head swinging are planned to further investigate the potential influence of such motion on the hydrodynamics and olfactory transport phenomena.

Finally, this study assumes that the flow in the nasal region is laminar, which cannot be justified *a priori* since the flow rate through the olfactory chamber is unknown. However, given CFD solutions, an *a posteriori* analysis was carried out to justify the laminar flow assumption. Specifically, results at the fastest simulated swimming speed (1.55 m/s) revealed that the maximum Reynolds number occurs within the incurrent nostril, where the value is approximately 1600, within the laminar regime for steady internal flow [31], [32]. Reynolds numbers within the sensory channels were much lower (in the 10–30 range), indicating low-Reynolds-number laminar flow in these passages.

### Boundary Conditions

The CFD domain was set up such that the reconstructed model was placed in a large computational box, and a uniform flow was imposed parallel to the body axis to model a swimming specimen. Based on the measured angle between the chord line of the cephalofoil and the oncoming flow direction, the angle of attack was approximately 0°. Experiments on juvenile scalloped hammerhead sharks (*Sphyrna lewini*) demonstrated average and maximum swimming speeds of 1.00 and 1.68 body lengths/sec, respectively [33]. Thus, given an estimated total length of 90 cm for the current specimen [3] and assuming a similar swimming performance for *Sphyrna tudes*, two CFD calculations were carried out with inflow velocities of 0.9 and 1.55 m/s to simulate average and maximum swimming speeds, respectively. Due to the bilateral symmetry of the model, a symmetry boundary condition was utilized along the medial plane of the head. Finally, no-slip boundary conditions were applied on all solid surfaces of the head and olfactory chamber, as previously justified (see Assumptions).

### Numerical Methods

The Semi-Implicit Method for Pressure-Linked Equations (SIMPLE) algorithm available in OpenFOAM was used to solve the incompressible continuity and Navier–Stokes equations governing steady, laminar flow. Iterative convergence of the SIMPLE solver was guaranteed by forcing the solution residuals to be less than approximately 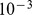. Additionally, various solution variables were monitored throughout the simulation to ensure convergence of the computed results. The computations were performed on 100 processors of a high-performance parallel computer cluster at Penn State University.

### Mesh Refinement Study

**Figure 8:**
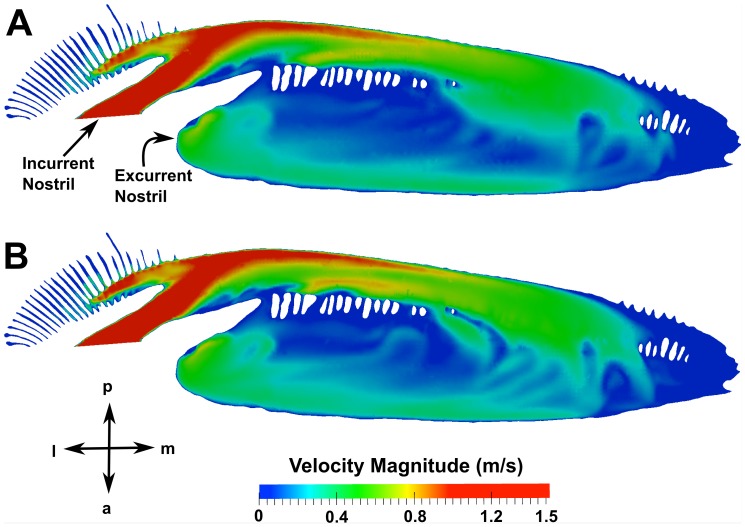
Qualitative mesh refinement results. Comparison of the calculated velocity field in the olfactory chamber for the (A) coarse and (B) fine meshes at a swimming speed of 1.55 m/s. a: anterior; l: lateral; m: medial; p: posterior.

A CFD mesh refinement study was carried out to verify the accuracy of the numerical solutions. Specifically, simulations were computed using both the coarse and fine computational meshes at the fastest swimming speed (1.55 m/s) to ensure mesh-independent CFD results. As shown in Figure 8, the qualitative features of the CFD solutions for the two meshes are consistent. That is, similar overall flow patterns, velocity distributions, pressure contours, etc. were obtained for both meshes. The only noticeable qualitative difference between the two solutions is that the results for the fine mesh contain smaller scales of motion compared to the coarse mesh solution. This is because the fine mesh is capable of resolving smaller laminar flow structures, the effect of which on the overall solution must be assessed quantitatively. Table 1 summarizes the quantitative differences between the coarse and fine mesh solutions. Specifically, percent differences were calculated for the pressure drop between the incurrent and excurrent nostrils and the flow rate through the olfactory chamber, which yielded values of 2.7% and 2.1%, respectively. Such small differences, along with the qualitative similarities between the two solutions, indicate that the fine mesh is sufficiently resolved and that the associated CFD solution is “mesh independent.”

**Table 1:** Quantitative mesh refinement results.

	Coarse Mesh	Fine Mesh	Percent Difference
Pressure Difference, Pa	502.06	515.87	2.7%
Olfactory Flow Rate, mL/s	7.86	8.03	2.1%

Quantitative comparison of the coarse and fine mesh solutions at a swimming speed of 1.55 m/s. The olfactory flow rate was calculated as the flow rate through the incurrent nostril. The pressure difference is taken as the difference in the mean pressures between the incurrent and excurrent nostrils.

## Results

From the micro-CT and MRI scans, and the resulting three-dimensional reconstruction of the anatomy, the present *Sphyrna tudes* specimen appears to be representative of the species in general. Specifically, the external and internal nasal morphology of the cadaver specimen compares well with brief descriptions of the nasal morphology of hammerhead sharks reported by others [1], [2], and with the internal nasal morphology of sharks in general [1], [2], [13], [20], [34], [35]. Thus, though slight intraspecies variability of the olfactory organ may occur, the gross morphology of the nasal region and the hydrodynamics of olfaction reported in this study are believed to be generally representative of *Sphyrna tudes.* Indeed, studying a range of specimens at this level of detail is not presently practical and is well beyond our scope. Rather, a detailed description of the form and function of the nasal region in a typical golden hammerhead shark (*Sphyrna tudes*) is provided.

### External Hydrodynamics

**Figure 9:**
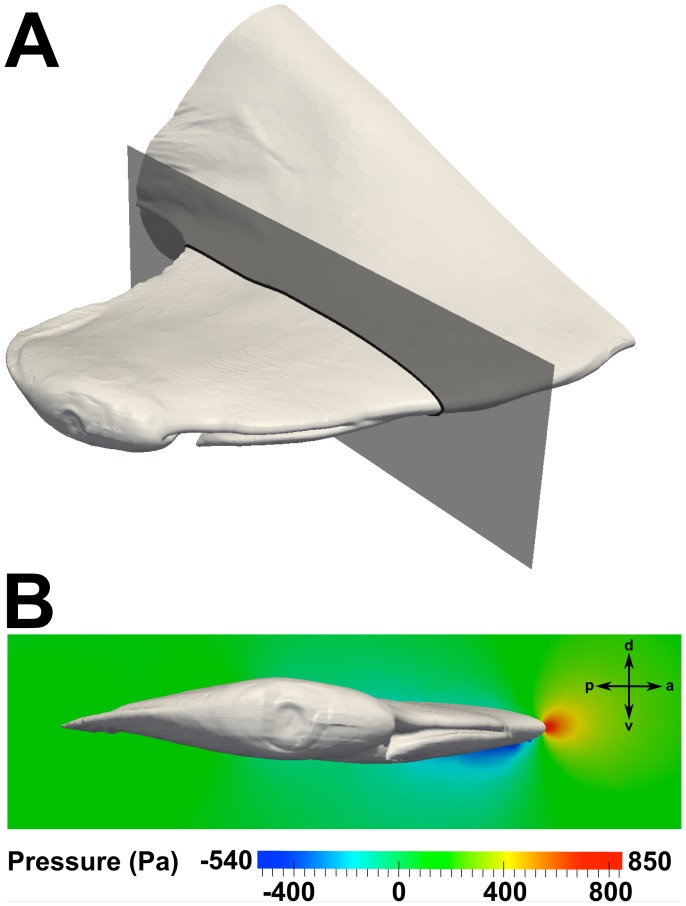
External pressure distribution. The pressure distribution on a plane at the spanwise location indicated in (A) is shown for a swimming speed of 1.55 m/s in (B). a: anterior; d: dorsal; p: posterior; v: ventral.

Several important results regarding the external hydrodynamics were extracted from the CFD simulations. First, the overall lift force was calculated by integrating the pressure and skin friction forces over the model. At the maximum and average swimming speeds, the lift force was calculated to be 1.82 N and 0.60 N, respectively, in the ventral direction. Figure 9 illustrates the pressure distribution around the cephalofoil. As shown in Figure 9B, the pressure on the ventral side of the head is lower than on the dorsal side of the head, resulting in a net force directed ventrally that increases with swimming speed.

**Figure 10:**
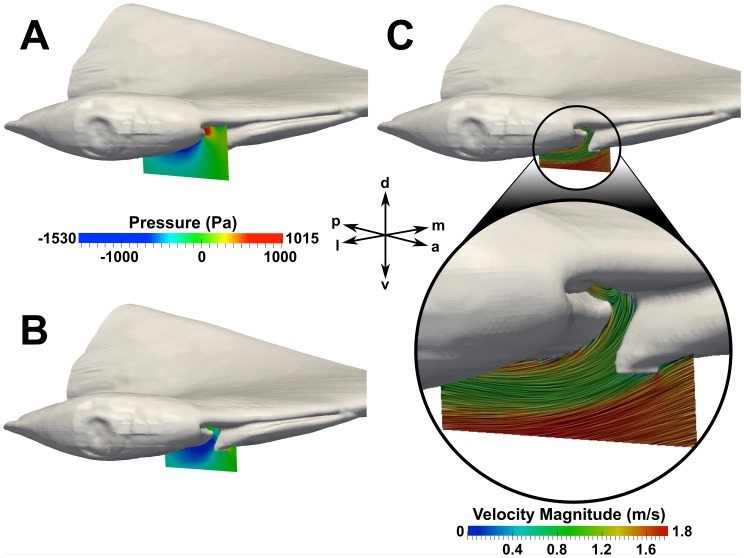
External flow in the nasal region. Pressure distributions at a swimming speed of 1.55 m/s are shown at spanwise locations near the (A) incurrent and (B) excurrent nostrils. Surface-limited streamlines and velocity contours are shown near the excurrent nostril in (C). a: anterior; d: dorsal; l: lateral; m: medial; p: posterior; v: ventral.

Figure 10 shows pressure distributions at the spanwise locations of the incurrent and excurrent nostrils, and flow patterns near the excurrent nostril. At the location of the incurrent nostril (Figure 10A), a flow stagnation point is shown to exist that results in a significantly higher pressure there compared to the flow exiting the excurrent nostril (Figure 10B). The streamlines in Figure 10C reveal that the flow at the lateral end of the major nasal groove impinges on the ventral lip of the incurrent nostril, where the flow stream splits and is directed either into the incurrent nostril or around the ventral side of the cephalofoil. The latter flow stream then accelerates due to the curvature of the cephalofoil, leading to the low pressure region seen in Figure 10B at the location of the excurrent nostril. It is this overall pressure difference between the incurrent and excurrent nostrils that induces flow through the olfactory chamber.

As summarized in Table 2, the present CFD simulations reveal that the pressure difference between the incurrent and excurrent nostrils, calculated as the difference in the average pressures (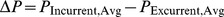), is 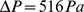 and 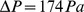 for the maximum and average swimming speeds, respectively. Additionally, the olfactory flow rate, taken as the flow rate through the incurrent nostril, for the maximum and average swimming speeds was calculated to be 8.0 mL/s and 4.5 mL/s, respectively. Thus, a faster swimming speed results in a greater pressure difference between the incurrent and excurrent nostrils, thereby inducing a larger olfactory flow rate.

**Table 2:** Overall pressure drop and flow rate through the olfactory chamber at each swimming speed.

	Average Speed	Maximum Speed
Pressure Difference, Pa	174	516
Olfactory Flow Rate, mL/s	4.5	8.0

Quantitative comparison of the CFD solutions at the maximum (1.55 m/s) and average (0.9 m/s) swimming speeds. The pressure difference was calculated as the difference in the mean pressures between the incurrent and excurrent nostrils. The olfactory flow rate was calculated as the flow rate through the incurrent nostril.

Additionally, as illustrated in Figures 3 and 10, the excurrent nostril is configured such that it protrudes below the ventral side of the head. At first glance, it may seem that the excurrent nostril would behave as a “blunt body” to the oncoming flow, thereby causing a low-pressure hydrodynamic wake to form downstream. However, as illustrated in Figure 10C, the fluid flowing out of the excurrent nostril prevents the formation of such a wake. Rather, flow leaving the excurrent nostril is directed over the ventral side of the cephalofoil, where it joins the ventral flow stream from the major nasal groove and subsequently accelerates due to the curvature of the head. This acceleration causes an associated decrease in pressure and the formation of a low-pressure region that augments the overall pressure difference between the incurrent and excurrent nostrils, and therefore the flow rate through the olfactory chamber.

**Figure 11:**
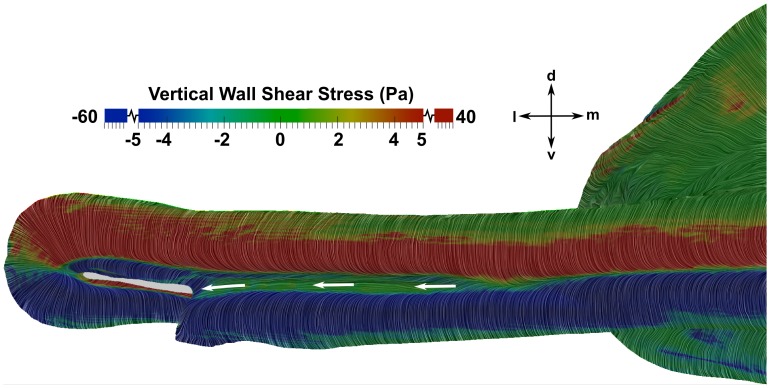
External flow patterns on the anterior edge of the head. Flow patterns over the cephalofoil at a swimming speed of 1.55 m/s are visualized using surface-limited streamlines colored by the vertical component of the wall shear stress, where positive values indicate dorsally-directed flow and negative values designate ventrally-directed flow. The white arrows illustrate the laterally-directed flow in the major nasal groove. d: dorsal; l: lateral; m: medial; v: ventral.

The role of the major nasal groove in directing oncoming flow was also investigated. Figure 11 shows surface-limited streamlines on the outer surface of the cephalofoil, colored by the vertical component of the wall shear stress. In this figure, positive values (red regions) indicate that the flow is directed dorsally, while negative values (blue regions) indicate ventrally-directed flow. As shown in Figure 11, the flow stagnates at the anterior edge of the head near the major nasal groove. Flow outside of the major nasal groove is directed dorsally and ventrally around the head, whereas flow inside the major nasal groove is directed laterally towards the incurrent nostril. Thus, as suggested in previous studies [1], [2] and observed by Abel et al. [3], the major nasal groove is responsible for directing oncoming flow towards the incurrent nostril. However, the present CFD simulations reveal that not all of the flow from the major nasal groove enters the incurrent nostril, as observed in Figure 10C and previously discussed. Specifically, the orientation of the major nasal groove relative to the incurrent nostril is such that some of the flow leaving the lateral end of the groove is directed over the ventral side of the head, away from the inlet naris. Thus, the morphology of the major nasal groove induces external flow patterns that extend the hydrodynamic “reach” of the incurrent nostril, enabling the shark to sample a wide spatial range, while limiting the incurrent olfactory flow rate.

**Figure 12:**
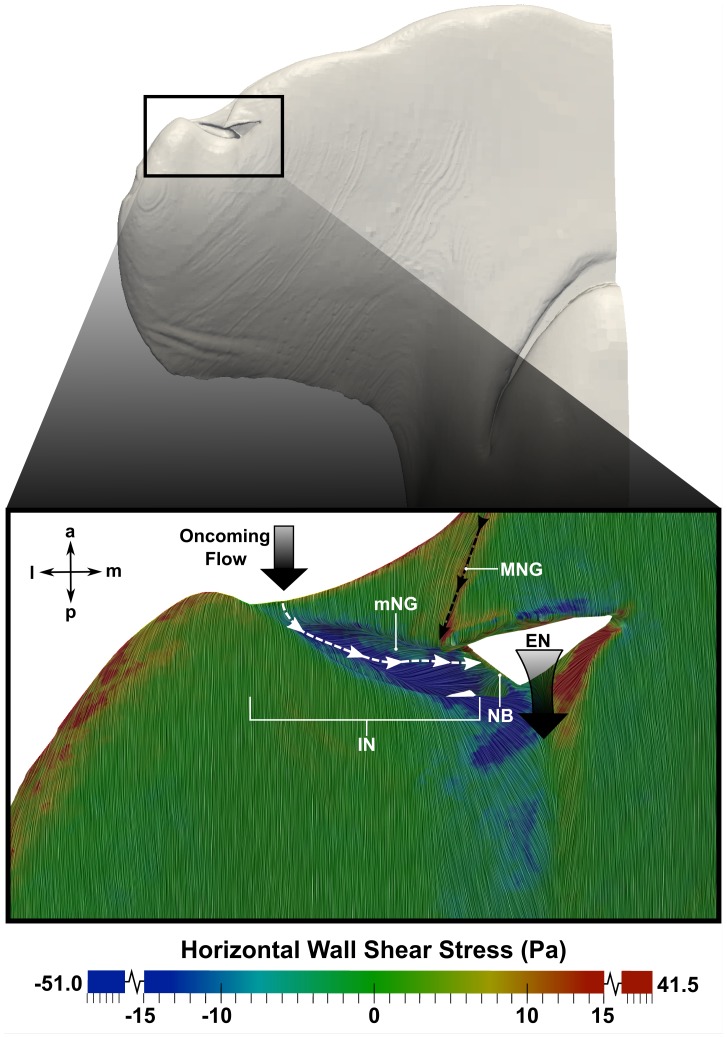
Flow in the minor nasal groove. Flow patterns in the minor nasal groove are visualized at a swimming speed of 1.55 m/s. Surface-limited streamlines are colored by the horizontal component of the wall shear stress; positive values indicate laterally-directed flow, while negative values indicate medially-directed flow. The large black arrows indicate the direction of the oncoming flow and the flow leaving the excurrent nostril. The black dotted line shows the flow path from the major nasal groove, while the white dotted line illustrates the flow path in the minor nasal groove. a: anterior; l: lateral; m: medial; p: posterior; mNG: minor nasal groove; MNG: major nasal groove; IN: incurrent nostril; NB: nasal bridge; EN: excurrent nostril.

**Figure 13:**
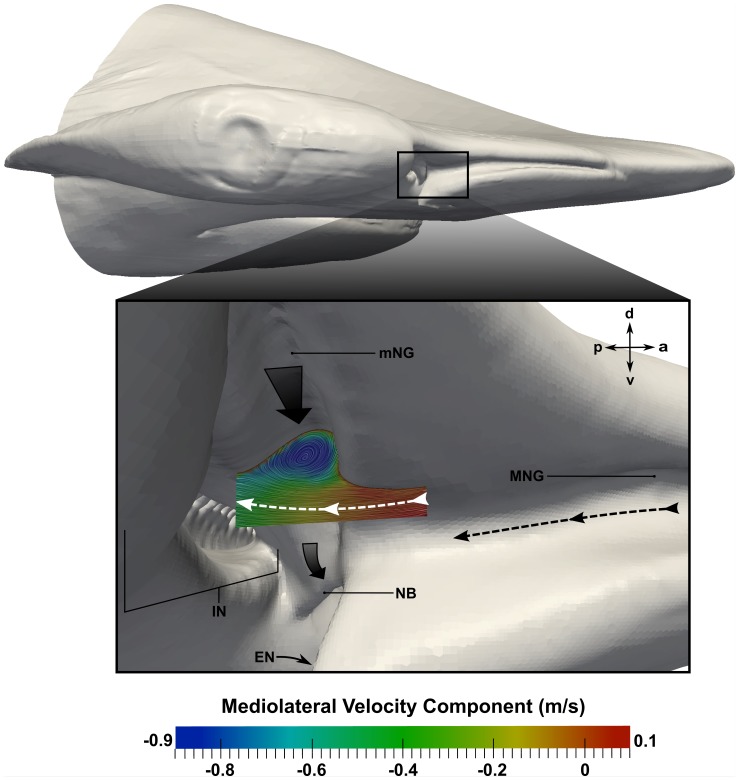
Flow in the minor nasal groove. Flow patterns in the minor nasal groove are visualized at a swimming speed of 1.55 m/s. Surface-limited streamlines are colored by the mediolateral component of velocity, where positive values indicate laterally-directed flow and negative values indicate medially-directed flow. The large black arrows show the primary flow direction in the minor nasal groove and over the nasal bridge. The black dotted line shows the flow path from the major nasal groove, and the white dotted line shows the flow path into the incurrent nostril. a: anterior; d: dorsal; p: posterior; v: ventral; mNG: minor nasal groove; MNG: major nasal groove; IN: incurrent nostril; NB: nasal bridge; EN: excurrent nostril.

The role of the minor nasal groove, first reported by Abel et al. [3], was also investigated. Figures 12 and 13 show surface-limited streamlines near the minor nasal groove, colored by either the horizontal component of the wall shear stress (Figure 12) or the mediolateral component of velocity (Figure 13). In both figures, negative values (blue regions) indicate medially-directed flow, and positive values (red regions) indicate laterally-directed flow. As shown in Figure 12, fluid enters the minor nasal groove predominantly from the oncoming flow stream. Following the white dotted line, flow in the minor nasal groove is directed medially towards the nasal bridge, which subsequently turns the flow towards the excurrent nostril where it is entrained by the excurrent flow stream (large black arrow in Figure 12) and directed over the ventral side of the cephalofoil. This general flow path is consistent with the observations made by Abel et al. [3].

Figure 13 illustrates additional flow patterns in the minor nasal groove. Surface-limited streamlines in a plane perpendicular to the minor nasal groove reveal that fluid flowing into the incurrent nostril (indicated by the white dotted line in Figure 13) flows over the minor nasal groove, which adds swirl to the medially-directed flow within the groove. Also note that the flow speed in the minor nasal groove (∼0.9 m/s) is relatively high compared to the free-stream velocity (1.55 m/s), indicating that there is a significant amount of medially-directed flow in the minor nasal groove. For comparison, flow rates in the major and minor nasal grooves were calculated at both swimming speeds, and are summarized in Table 3. At the average swimming speed, the flow rate in the minor nasal groove is 0.32 mL/s, compared with 1.3 mL/s in the major nasal groove. Likewise, at the maximum swimming speed, the flow rate in the minor nasal groove is 0.54 mL/s, compared with 2.3 mL/s in the major nasal groove. Thus, at both swimming speeds the flow rate in the major nasal groove is approximately four times larger than that in the minor nasal groove. Further, given that the olfactory flow rate (the flow rate through the incurrent nostril) at the average and maximum swimming speed is 4.5 mL/s and 8.0 mL/s, respectively, in each case the minor nasal groove redirects approximately 7% of the oncoming flow away from the incurrent nostril.

**Table 3:** Flow rates in the major and minor nasal grooves.

	Average Speed	Maximum Speed
Major Nasal Groove Flow Rate, mL/s	1.3	2.3
Minor Nasal Groove Flow Rate, mL/s	0.32	0.54

Quantitative comparison of the flow rates in the major and minor nasal grooves at the maximum (1.55 m/s) and average (0.9 m/s) swimming speeds.

Thus, both the major and minor nasal grooves are configured such that they direct some flow away from the incurrent nostril, thereby limiting the flow rate through the olfactory chamber, which may serve to protect the delicate lamellae. Additionally, the major nasal groove extends the hydrodynamic reach of the inlet naris by directing a portion of the oncoming flow towards the incurrent nostril, thereby permitting the shark to sample a larger spatial extent. Given these hydrodynamic results, it is clear that the external morphology of the nasal region of the hammerhead shark likely confers a chemosensory advantage over non-sphyrnid species.

### Internal Hydrodynamics

**Figure 14:**
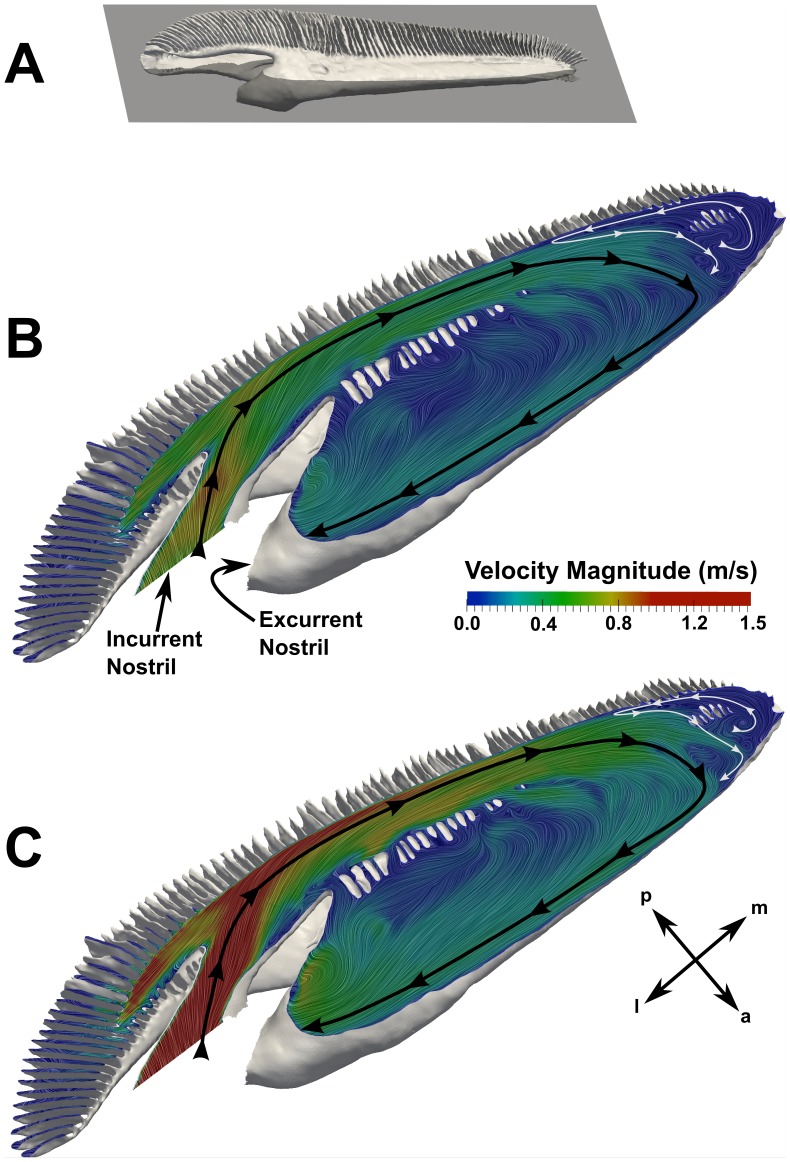
Internal flow patterns. Surface-limited streamlines and velocity contours in the olfactory chamber are shown on a plane (A) perpendicular to the dorsoventral axis that passes through the apical gap between the upper and lower lamellar arrays. Flow patterns are shown for the (B) average and (C) maximum swimming speed cases. The black and white lines illustrate the overall flow patterns within the olfactory chamber. a: anterior; l: lateral; m: medial; p: posterior.

Internally, the flow through the olfactory chamber is complex and highly three-dimensional. Figure 14 illustrates the overall flow path within the olfactory chamber at both swimming speeds. These flow patterns were extracted from the CFD results in a plane perpendicular to the dorsoventral axis that passes through the apical gap between the upper and lower lamellar arrays. As shown by the solid black lines, water enters the olfactory chamber via the incurrent nostril and flows medially through the incurrent channel, where it subsequently turns and flows laterally through the excurrent channel and exits the olfactory chamber via the excurrent nostril. Comparing Figures 14B and 14C, these overall flow patterns are remarkably similar for both swimming speeds. In both cases a near-stagnant recirculation region is observed at the medial end of the olfactory chamber (illustrated by solid white lines). Hydrodynamically, this reversed flow region is due to the relatively sharp hairpin bend at the medial end of the chamber, where an adverse pressure gradient pushes against the oncoming flow, leading to low-speed reversed flow and mixing in the corner. The only significant difference in the overall flow patterns between the average and maximum swimming speed cases is the size of the recirculation region; a larger recirculation region is shown to exist at the slower swimming speed. At faster swimming speeds, the incoming flow penetrates deeper into the olfactory chamber due to the increased momentum of the fluid, resulting in a smaller recirculation zone at the medial end of the chamber and, consequently, a larger functional region for olfaction. This observation was similarly noted by Abel et al. [3], though their flow visualization experiments were conducted at lower flow speeds (less than the estimated physiological range for the *Sphyrna tudes* specimen) and utilized a low-resolution plastic model (see Introduction).

**Figure 15:**
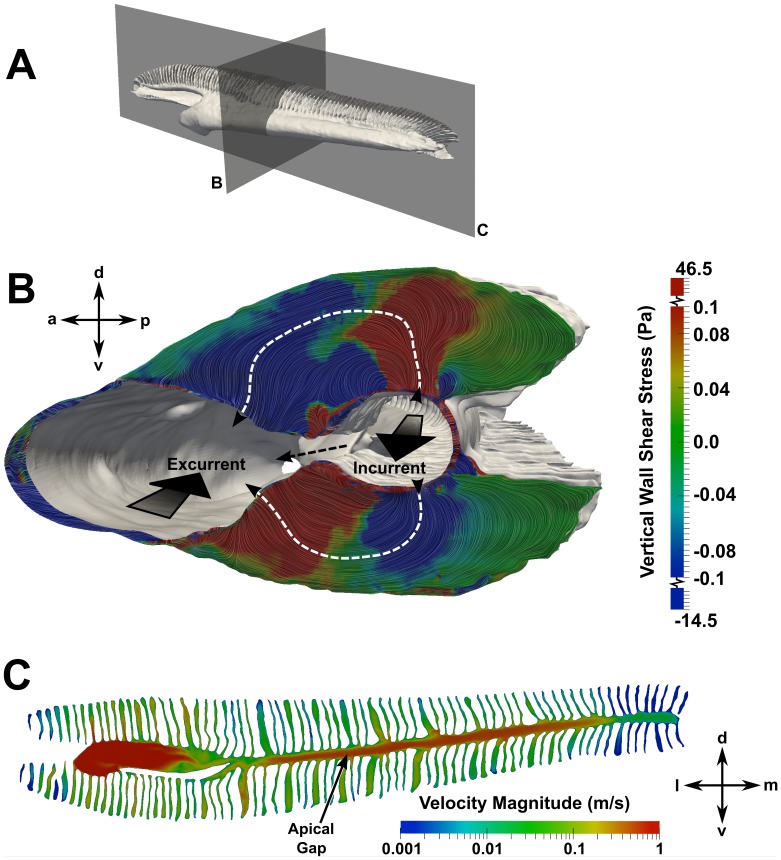
Flow through the apical gap and sensory channels. Flow patterns in the apical gap and sensory channels are visualized on planes at the locations indicated in (A). In (B), surface-limited streamlines and contours of the vertical component of the wall shear stress are used to visualize flow patterns within the sensory channels at a swimming speed of 1.55 m/s. The large black arrows illustrate the flow direction in the incurrent and excurrent channels. The dashed black line shows the flow path through the apical gap between the upper and lower lamellar arrays, and the dashed white lines illustrate the flow paths through the sensory channels. Positive values of shear stress indicate dorsally-directed flow, and negative values designate ventrally-directed flow. In (C), velocity contours on a slice through the apical gap show a relative comparison of the flow speeds in the apical gap and sensory channels. a: anterior; d: dorsal; l: lateral; m: medial; p: posterior; v: ventral.

Flow within the incurrent channel can reach the excurrent channel in one of two ways: 1) the apical gap between the dorsal and ventral lamellar arrays or 2) through the sensory channels. Figure 15B illustrates these flow paths, where the dashed black line indicates the apical gap route (which was shown in Figure 14), and the dashed white lines illustrate the flow paths through the sensory channels, which are lined with olfactory sensory epithelium. Functionally, the apical gap route is a partial bypass that limits the flow rate of fluid through the sensory channels, which may serve to protect the delicate lamellae and sensory epithelium [12]. Quantitatively, the present CFD results reveal that, at the average swimming speed, approximately 46% of the water entering the olfactory chamber flows through the sensory channels; at the maximum swimming speed, approximately 55% of the incoming water flows through the sensory channels. The remaining fluid is bypassed from the incurrent channel to the excurrent channel via the apical gap. In Figure 15B, flow through the sensory channels is visualized by surface-limited streamlines and contours of the vertical component of the wall shear stress, which reveals that there is flow over the entire surface of the upper and lower lamellae. However, compared with flow through the apical gap, flow speeds within the sensory channels are much lower (∼1 m/s versus ∼0.1 m/s, respectively), as shown in Figure 15C.

**Figure 16:**
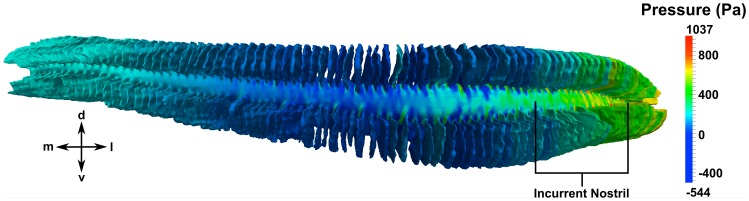
Internal pressure distribution. A posterior view of the olfactory chamber shows the pressure distribution along the length of the incurrent channel. d: dorsal; l: lateral; m: medial; v: ventral.

**Figure 17:**
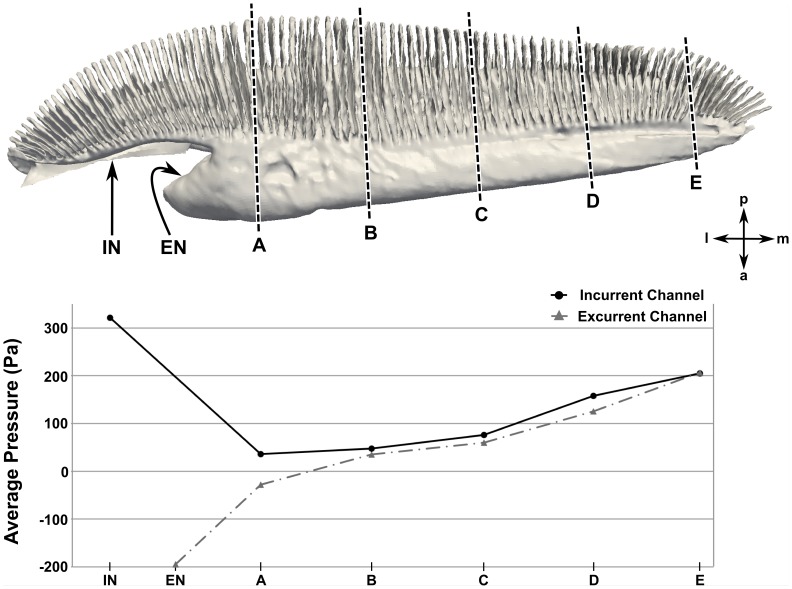
Pressure distribution along the incurrent and excurrent channels. The average pressure in the incurrent and excurrent channels at the maximum swimming speed (1.55 m/s) is plotted at various locations along the length of the olfactory chamber. IN: incurrent nostril; EN: excurrent nostril; a: anterior; l: lateral; m: medial; p: posterior.

Finally, given the near-stagnant recirculation region at the medial end of the olfactory chamber, we investigate the internal pressure and flow distribution within the nasal passages. As shown in Figure 16, the largest pressure gradient occurs near the incurrent nostril. The pressure is then fairly uniform in the center of the olfactory chamber before it gradually increases near the medial end, where the flow reverses direction. Figure 17 shows the average pressure in the incurrent and excurrent channels along the length of the olfactory chamber at the maximum swimming speed. Most importantly, this plot illustrates the pressure difference between the incurrent and excurrent channels, which is the mechanism that drives flow through the sensory channels. Accordingly, the relatively large pressure difference at location A (compared to locations B-E) indicates that the sensory channels in this region see larger flow rates than those near the medial end of the chamber, where the pressure difference is comparatively smaller.

**Figure 18:**
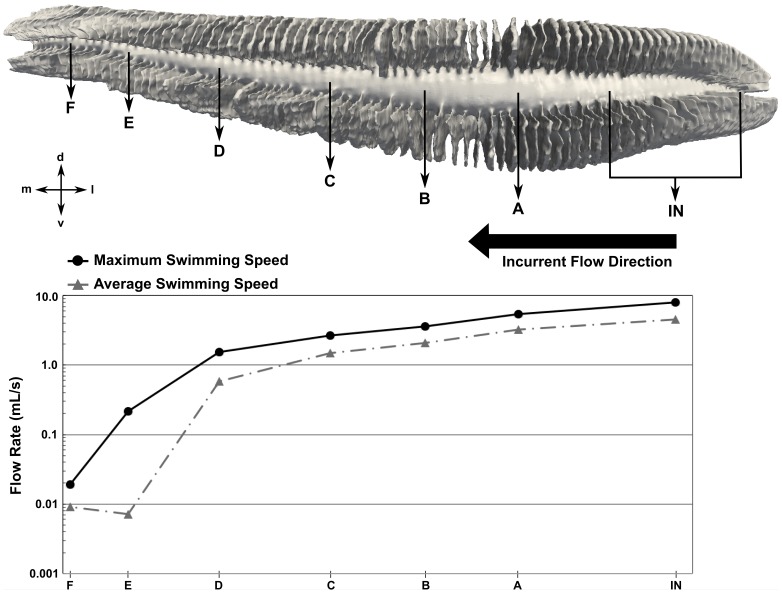
Mediolateral flow distribution in the olfactory chamber. The flow rate along the incurrent channel is plotted at various locations for both swimming speeds. The large black arrow shows the incurrent flow direction. d: dorsal; l: lateral; m: medial; v: ventral.

To quantify the flow distribution within the olfactory chamber, the flow rate along the incurrent channel was calculated at various locations, shown in Figure 18 for both swimming speeds. The dip at location E for the average swimming speed is due to the larger recirculation region observed in Figure 14. As shown, at both swimming speeds the flow rate decreases dramatically along the length of the incurrent channel as fluid passes into the excurrent channel via either the apical gap or the sensory channels. Consequently, the flow rates within the sensory channels also decrease medially along the olfactory chamber. That is, the medial lamellae receive much less fluid and experience lower flow speeds than those near the incurrent nostril.

## Discussion

The present results have led to several interesting observations regarding the hydrodynamics of olfaction in the hammerhead shark *(Sphyrna tudes*). At 0° angle of attack, the cephalofoil was shown to produce a relatively small (∼1 N) ventrally-directed lift force at physiologically realistic swimming speeds. Thus, the unique head morphology of the hammerhead shark confers several chemosensory advantages while having little overall hydrodynamic impact on the fish (although, at larger angles of attack, the lift force will be greater and may appreciably affect the overall hydrodynamics). Also, as previously noted [2]–[5], the hammerhead benefits from a wide lateral separation between its olfactory organs, which may be used for enhanced olfactory tropotaxis. Moreover, this study has shown that the external hydrodynamics of the cephalofoil impart several additional chemosensory advantages:

- The incurrent and excurrent nostrils are located in regions of high and low pressure, respectively, resulting in an overall pressure difference that induces flow through the olfactory chamber (see Figure 10). The incurrent nostril is located at the anterior edge of the cephalofoil, where the maximum pressure occurs at the flow stagnation point. The excurrent nostril is located near the ventral side of the head, where the flow accelerates due to the curvature of the cephalofoil, causing an associated decrease in pressure and the formation of a low-pressure region.
- The major (prenarial) nasal groove that extends medially from the incurrent nostril, along the anterior edge of the cephalofoil, directs some flow into the incurrent nostril (Figures 10 and 11), as previously proposed [1]–[3]. This enables the shark to sample both a larger volume of fluid and a wider spatial range. However, not all of the flow from the major nasal groove enters the incurrent nostril; a significant portion is directed away from the inlet. Thus, the morphology of the major nasal groove induces external flow patterns that extend the hydrodynamic reach of the incurrent nostril, while limiting the incurrent olfactory flow rate.

Internally, flow through the olfactory chamber was investigated, where the following overall flow path was illustrated: incurrent nostril 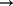 incurrent channel 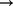 sensory channels *or* apical gap 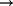 excurrent channel 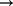 excurrent nostril (see Figures 14 and 15). At faster swimming speeds the incoming flow was shown to penetrate deeper into the olfactory chamber, resulting in a smaller near-stagnant recirculation region at the medial end of the chamber. This implies a larger functional region for olfaction at faster swimming speeds, though slower swimming speeds may also enhance chemical detection by facilitating longer odorant residence times in the olfactory chamber. Investigating such trade-offs requires simulation of odorant mass transport phenomena, which is a topic of future work.

As water circulates through the olfactory organ, we found that the flow rate decreases medially along the length of the chamber. As a result, the medial sensory channels receive much less fluid and experience lower flow speeds than those near the incurrent nostril. Functionally, this means that less odorant is delivered to the sensory channels at the medial end; however, lower flow speeds indicate a longer residence time for odorant deposition in the medial channels, which may enhance detection of some chemicals. Future odorant transport simulations are planned to investigate such functional trade-offs and the implications for olfaction.

The present study also revealed that there are multiple flow regulation mechanisms in the nasal region of the hammerhead shark that limit the flow rate of fluid through the sensory channels of the olfactory chamber. Externally, the major and minor nasal grooves direct some flow away from the incurrent nostril, thereby limiting the overall flow rate through the olfactory organ. Internally, the apical gap between the dorsal and ventral lamellar arrays allows a significant amount of fluid to bypass the sensory channels, providing an internal mechanism for limiting the flow rate of fluid between the lamellae. Specifically, at the average and maximum swimming speeds, approximately 46% and 55% of the incoming flow passes through the sensory channels, respectively, while the remaining flow bypasses the olfactory lamellae via the apical gap (see Internal Hydrodynamics). Thus, as the shark swims faster, a larger percentage of the fluid entering the olfactory chamber flows through the sensory channels, where chemical detection occurs. Even so, at the maximum swimming speed nearly half of the internal flow bypasses the sensory channels. Such external and internal flow regulation mechanisms that limit flow through the sensory channels likely function to protect the delicate lamellae and olfactory sensory epithelium, as previously suggested [3], [12].

Finally, given the morphological similarity of the olfactory chamber in *Sphyrna tudes* and many other sharks (see [1], [11]–[13], [15]), similar overall internal hydrodynamic flow patterns might be expected in these species, particularly those that possess an apical gap between their dorsal and ventral lamellar arrays (e.g., *Carcharhinus amblyrhynchos*, *Carcharhinus amboinensis*, *Hemipristis elongata*, *Hemiscyllium ocellatum*, *Sphyrna lewini* [11]). However, when externally compared with other shark species, hammerheads possess a broad, flat head and a wider lateral separation between olfactory organs that facilitates enhanced bilateral sampling for olfactory tropotaxis [2], [4], [5]. Moreover, there are interspecies differences in nasal morphology associated with the incurrent and excurrent nostrils and the presence (or absence) of major and minor nasal grooves. Compared with other species, hammerhead sharks possess a slit-like incurrent nostril, presumably a consequence of their flattened heads. Such differences in incurrent nostril shape (and size) could potentially influence the incurrent olfactory flow rate. Further, hammerhead sharks possess major and minor nasal grooves, whereas other sharks do not. Specifically, all eight species of hammerhead shark possess a minor nasal groove and four hammerhead species (including *Sphyrna tudes*) possess a major nasal groove [3]. Given that the major and minor nasal grooves were shown to significantly influence the external hydrodynamics of olfaction in the nasal region of *Sphyrna tudes*, these external features likely confer a chemosensory advantage over non-sphyrnid species. For example, the hydrodynamic reach of the incurrent nostril is likely to be much larger in hammerhead sharks that possess a major nasal groove, which allows these species to sample a larger spatial extent compared with sharks that lack a major nasal groove. Thus, the results of this study demonstrate the functional significance of the hydrodynamics of olfaction in the hammerhead shark (*Sphyrna tudes*), and further motivate the need for a comparative study of olfactory transport phenomena across a range of shark species.
